# Direct Multi-Material Reconstruction via Iterative Proximal Adaptive Descent for Spectral CT Imaging

**DOI:** 10.3390/bioengineering10040470

**Published:** 2023-04-12

**Authors:** Xiaohuan Yu, Ailong Cai, Ningning Liang, Shaoyu Wang, Zhizhong Zheng, Lei Li, Bin Yan

**Affiliations:** Henan Key Laboratory of Imaging and Intelligent Processing, PLA Strategic Support Force Information Engineering University, Zhengzhou 450001, China

**Keywords:** spectral computed tomography, image reconstruction, one-step material decomposition, iterative proximal adaptive descent

## Abstract

Spectral computed tomography (spectral CT) is a promising medical imaging technology because of its ability to provide information on material characterization and quantification. However, with an increasing number of basis materials, the nonlinearity of measurements causes difficulty in decomposition. In addition, noise amplification and beam hardening further reduce image quality. Thus, improving the accuracy of material decomposition while suppressing noise is pivotal for spectral CT imaging. This paper proposes a one-step multi-material reconstruction model as well as an iterative proximal adaptive decent method. In this approach, a proximal step and a descent step with adaptive step size are designed under the forward–backward splitting framework. The convergence analysis of the algorithm is further discussed according to the convexity of the optimization objective function. For simulation experiments with different noise levels, the peak signal-to-noise ratio (PSNR) obtained by the proposed method increases approximately 23 dB, 14 dB, and 4 dB compared to those of other algorithms. Magnified areas of thorax data further demonstrated that the proposed method has a better ability to preserve details in tissues, bones, and lungs. Numerical experiments verify that the proposed method efficiently reconstructed the material maps, and reduced noise and beam hardening artifacts compared with the state-of-the-art methods.

## 1. Introduction

Spectral computed tomography (spectral CT) has promising potentials in wide applications due to its ability to discriminate quantitative material for diagnostics and therapy evaluation in medical imaging [1,2]. Pieces of evidence are being found indicating that spectral CT can help improve the diagnosis of coronavirus disease (COVID-19) [3,4]. As one of the typical implementations of spectral CT, the principle of dual-energy CT (DECT) has been studied for a long time. Recent developments in energy-selective detectors have spurred research in this area, especially the improvement of photon-counting detectors (PCDs) [5]. However, the low signal-to-noise ratio (SNR) measurements, caused by pile-up, fluorescence effect, charge sharing, and photon scattering, affect the image quality and the precision of material decomposition [6]. Therefore, how to improve the accuracy of material decomposition while maintaining image quality is crucial to the field of medical imaging.

In recent years, there are two categories of methods for reconstructing material-specific images: two-step methods and one-step methods. Furthermore, the two-step methods can be divided into image domain- and projection domain-based methods. The image domain-based approaches [7,8,9,10] first reconstruct CT images from the polychromatic projection data and then obtain the corresponding material images by a decomposition step on CT images. Unfortunately, the quality of material results is always severely affected by beam-hardening artifacts and noise explosions caused by direct matrix inversion-based decomposition, especially when there are more than two materials to be separated [11]. On the other hand, for projection domain-based methods, multi-energy projections are first separated or decomposed into material-specific projections and then reconstructed through traditional algorithms [12,13,14]. However, it requires the projection to be in multi-energy, measured under strictly consistent and identical imaging geometry (i.e., the same source, object, and detector positions), which limits its application in fast kVp switching [15] or multi-source-multi-detector [16] systems. In addition, the material-specific results of two-step methods are dependent on the quality of the first step, and it is difficult for the second step to compensate for the errors caused by the first step.

To avoid these problems for dual material imaging in dual spectral CT, several methods have been proposed, aimed at directly obtaining material-specific images from the nonlinear observation measurements, called the abovementioned one-step methods. All these one-step methods are iterative, combing forward models of the reconstruction with the material separation process. For example, Zhao et al. utilized the first-order Taylor expansion of nonlinear observations and proposed an extended algebraic reconstruction technique (EART) [17] for DECT. For the consideration of the convergence efficiency, couple variants of EART have been proposed, e.g., the simultaneous EART (ESART) [18], the oblique projection modification technique [19], and the monochromatic images guided iteration method [20] in recent years. In addition, as the photons emitted by the X-ray source contain continuous energy distribution, and it is empirically assumed that the attenuated photons received by the detector follow the Poisson distribution, different statistical-based iterative methods have emerged. Xu et al. [21] developed a penalized likelihood algorithm to implement the decomposition of the basis materials for DECT. Long et al. [22], Weidinger et al. [23], and Mechlem et al. [24] designed the separable quadratic surrogates of spectral CT statistical models to achieve the one-step material decompositions. Barber et al. [25] applied a primal-dual prototype framework [26] to the material imaging of spectral CT. Very recently, they further proposed investigating the convergence theory of the non-convex alternating direction of multipliers method (NcADMM) [27] and conducted the reconstruction of the PCD system to reduce beam hardening and metal artifacts [28].

Moreover, due to the inherent ill-conditioning of the CT inverse problem, it is often necessary to incorporate prior knowledge as a regularization term to suppress the noise of basis material images. To further enhance the quality of reconstruction, sparsity-based methods have been employed. For example, Cai et al. [29] adopted the Huber function [30] as the regularization term in a Bayesian approach. Chen et al. [31,32] applied the convex indicator function of the gradient image to enforce an upper bound on the material images and the monochromatic images. Zhang et al. [33] proposed a direct material reconstruction method that combined a total variation (TV) term with block matching and a 3D filtering term for DECT. However, for the above one-step iterative method, the convergence of algorithms based on the primal-dual framework is not satisfying, and some methods are even susceptible to noise due to a lack of the ability of noise suppression or the need to adjust the parameters of the regularization terms manually when the number of materials increases. Direct extensions and applications of the abovementioned one-step methods to multi-material reconstructions are unstable or will even cause failure, due to the increase in the ill-posedness from dual material to multi-material applications. Therefore, it is a key issue to design an efficient and accurate one-step method based on an appropriate optimization model for multi-material imaging in spectral CT.

In this work, for multi-material reconstruction, a direct one-step method is proposed, which combines material image regularization and data fidelity. A proximal step and an adaptive descent step are designed under the operator splitting framework. The convergence of the iterative algorithm is further analyzed to illustrate the theoretical effectiveness and stability of the new method. In order to verify the practical performance of the presented method, a series of numerical experiments have been conducted and shown that the proposed algorithm has improved results of noise suppression and beam-hardening elimination compared with image-domain based decomposition method and the state-of-the-art one-step material reconstruction methods.

The organization of this paper is as follows. Section 2 introduces the physical model, and describes the proposed reconstruction theory, as well as the convergence analysis of the established algorithm. Section 3 presents the numerical verifications of the proposed method and experimental comparisons with typical competing methods. The discussions and conclusions are subsequently presented in Section 4 and Section 5, respectively.

## 2. Materials and Methods

### 2.1. Multi-Material, Non-Linear forward Projection Model

The photon intensity of the rays emitted from the X-ray source measured by the detector will be attenuated when passing through the unknown object. The attenuation coefficients of the object f for the photon with energy E at position x can be expressed as f(x,E). The measurements can be simplified to Beer’s law under the assumption of a monochromatic beam $s_{j}=S_{j}(E)$ along the j-th ray as

(1) $$p_{j}=s_{j}\exp(-\int_{\ell_{j}}f(x,E)d\ell ),\;(j=1,\ldots ,J),$$

where $S_{j}(E)$ is the spectrum distribution of the photon intensity with energy E, $\ell_{j}$ is the integral line indexed by j, and J is the total counts of measured rays. First, represent the discretization of f(x,E) as $f_{E}={\left[f_{1}{}_{,E},f_{2}{}_{,E},\ldots ,f_{I,E}\right]}^{T}$, where I is the total number of voxels and ${\left[\cdot \right]}^{T}$ denotes the transpose of a vector. With the discretization of the variables, the measurements of (1) can be further expressed in a discretized form as

(2) $$p_{j}=s_{j}\exp(-{\left[Af_{E}\right]}_{j}),\;(j=1,\ldots ,J),$$

where ${\left[Af_{E}\right]}_{j}=\sum_{i=1}^{I}a_{ji}f_{i}$ represents the j-th line integral. Matrix $A=[a_{ji}]\in R^{J\times I}$ represents the X-ray transform, and its element $a_{ji}$ denotes the contribution of the i-th pixel to the projection along the j-th X-ray path.

The unknown image $f_{E}$ often contains multiple materials (saying K kinds of materials), where the spatial distribution of the fraction of each material is of interest. In the presented work, the X-ray spectrum S(E) is equally discretized into M segments with an energy interval of $\delta_{E}$ such that $S_{m}=S(m\cdot \delta_{E})$, m=1,2,…,M. The monochromatic image $f_{m}$ at a specific energy $E=m\cdot \delta_{E}$ can be decoupled into energy-dependent terms $\mu_{mk}=\mu_{mk}(E)$, k=1,2,…,K, and spatial-dependent terms $b_{k}=b_{k}(x)\in R^{I}$. Therefore, the monochromatic image $f_{m}$ can be expressed by the linear combination of energy-dependent and spatial-dependent terms as

(3) $$f_{m}(b)=\sum_{k=1}^{K}\mu_{mk}b_{k},\;(m=1,2,\ldots ,M),$$

where $\mu_{mk}$ are the known attenuation coefficients of the basis material $b_{k}$ at energy m. Concatenating the K kinds of basis materials in the form of $b={\left(b_{1}{}^{T},b_{2}{}^{T},\ldots ,b_{K}{}^{T}\right)}^{T}$, the image $f_{m}(m=1,2,\ldots M)$ to be reconstructed is tantamount to that of the basis images b.

For spectral CT imaging, consider that there are S energy spectra in total, and the expected number of photon counts $p_{sj}(b)$ at spectrum s after the j-th ray penetrating the unknown object is given by the following non-linear model when taking the negative logarithm

(4) $$\begin{array}{l} p_{sj}(b)=-\ln\sum_{m=1}^{M}S_{sjm}\exp(-\sum_{k=1}^{K}\mu_{mk}{\left[Ab_{k}\right]}_{j}),\\ s=1,2,\ldots ,S,\\ j=1,2,\ldots ,J, \end{array}$$

where $S_{sjm}=S_{sjm}(E)\cdot \delta_{E}$ is the normalized X-ray spectrum s at energy m for the j-th ray, i.e., $\sum_{m=1}^{M}S_{sjm}=1$. For compact forms, rearrange the measured data and the non-linear data term into the form of the vectors $P^{M}=\left(p_{1}^{M};p_{2}^{M};\ldots ;p_{S}^{M}\right)\in R^{JS}$ and $P(b)=\left(p_{1}(b);p_{2}(b);\ldots ;p_{S}(b)\right)\in R^{JS}$, respectively, where $p_{s}^{M},p_{s}(b)$ denote the vectors of size J, with elements of $p_{sj}^{M}$ and $p_{sj}(b)$, respectively.

### 2.2. Optimization Model and Algorithm

The ill-posedness of Equation (4) will increase as the number of materials grows. Moreover, noise and inconsistency in the measurements also increase the difficulty of solving Equation (4) directly. Therefore, the sparsity-based prior is introduced into the one-step reconstruction model

(5) $$\underset{b}{\min}\frac{1}{2}{\left\|P\left(b\right)-P^{M}\right\|}_{2}^{2}+\lambda {\left\|\nabla b\right\|}_{1},$$

where $\lambda =[\lambda_{1},\ldots ,\lambda_{K}]$ denote the regularization parameters of different basis materials.

To make the following statements more concise, here we use some symbols to represent the fidelity and regularization terms in the model, i.e., $G(b)=\frac{1}{2}{\left\|P(b)-P^{M}\right\|}_{2}^{2}$ and $B(Kb)={\left\|Kb\right\|}_{1}$, where K is related to the finite difference operator—in this model. However, the function G(b) is non-convex with respect to the variable b, and the relevant algorithms of convex optimization cannot be applied directly. Therefore, P(b) is linearized by using the first-order Taylor expansion such that G(b) is convex about b. Furthermore, the regularization term B(Kb) is a non-smooth function, and the first derivation of the objective function in the problem (5) does not exist at some point. Based on these concepts, we design a new iterative algorithm according to the first-order optimality condition of the optimization problem. The algorithm consists of two main steps: the proximal step and the descent step. The former is used to obtain the reconstructed material images, and the latter determines the direction of the updating.

Specifically, the concept of sub-differential is further applied to obtain the first-order optimal condition of the optimization problem above as follows:

(6) $$0\in \partial G(b)+\lambda^{T}K^{T}\partial B(\lambda Kb),$$

where the symbol “∈” means the sub-gradient q of a closed proper convex function Q that satisfies ∂Q(x):={q:Q(x)−Q(y)≥〈q,x−y〉,∀y}. In addition, if Q(⋅) is further continuously differentiable, then there is only one gradient in the set of sub-differential ∂Q(x), that is ∂Q(x)={∇Q(x)}. Then, introduce an auxiliary variable y to split the condition (6) in the following form:

(7) $$\{\begin{array}{l} 0\in \partial G(b)+\lambda^{T}K^{T}y,\\ 0\in \partial B(\lambda Kb)-y. \end{array}$$

Assuming the current iteration points $\left(b^{\left(n\right)},y^{\left(n\right)}\right)$ are known, we use the idea of forward–backward splitting to obtain the iterative scheme

$$\begin{array}{c} \alpha b^{\left(n\right)}-\lambda^{T}K^{T}y^{\left(n\right)}\in (\alpha I+\partial G)(b),\\ y^{\left(n\right)}+\beta \lambda Kb^{\left(n\right)}\in (\beta I+\partial B)(v)\;v=\lambda Kb, \end{array}$$

where α,β are nonnegative parameters. The unique solution exists due to the linearization of G(⋅) and the properties of B(⋅). In addition, to maintain the stability of the algorithm, the new iteration is combined with the results of the previous step. Therefore, the designed proximal step contains the following three equations:

(8) $$u^{\left(n\right)}={\left(\alpha I+\partial G\right)}^{-1}(\alpha b^{\left(n\right)}-\lambda^{T}K^{T}y^{\left(n\right)})=\underset{u}{\arg\min}\{G(u)+\frac{\alpha }{2}{\left\|u+\frac{\lambda^{T}K^{T}y^{\left(n\right)}}{\alpha }-b^{\left(n\right)}\right\|}_{2}^{2}\},$$

(9) $${\hat{y}}^{\left(n\right)}=(1-t)b^{\left(n\right)}+tu^{\left(n\right)},$$

(10) $$\begin{array}{l} v^{\left(n\right)}={\left(\beta I+\partial B\right)}^{-1}(y^{\left(n\right)}+\beta \lambda K{\hat{y}}^{\left(n\right)})\\ =\underset{v}{\arg\min}\{B(v)+\frac{\beta }{2}{\left\|v-\lambda K{\hat{y}}^{\left(n\right)}-\frac{y^{\left(n\right)}}{\beta }\right\|}_{2}^{2}\}, \end{array}$$

where t is the parameter introduced to ensure that no abnormal point occurs.

The proximal step is one of the key steps in the material reconstruction model. It contains the forward transmission and the back projection in Equation (8). This paper utilized the multi-material ESART algorithm [18,33] to solve the proximal step and adopt the ordered subsets (OS) technique [34] to accelerate the algorithm; the detailed formulas are given in the Appendix A. Equation (10) is solved by a soft-shrinkage operator

(11) $$v^{\left(n\right)}=shrink(\lambda K{\hat{y}}^{\left(n\right)}+\frac{y^{\left(n\right)}}{\beta },\frac{1}{\beta }),$$

where $shrink(v,r)=sign(v)\max\{\left|v\right|-\frac{1}{r},0\}$.

Regarding the descent step, there are two variables in conditions (7) that need to be updated, i.e.,

(12) $$b^{\left(n+1\right)}=b^{\left(n\right)}-\gamma_{\left(n\right)}d_{1}{}^{\left(n\right)},$$

(13) $$y^{\left(n+1\right)}=y^{\left(n\right)}-\gamma_{\left(n\right)}d_{2}{}^{\left(n\right)},$$

where $\gamma_{\left(n\right)}$ denotes the step size to update the new iteration point adaptively, and its calculation is expressed as

(14) $$\gamma_{\left(n\right)}=\theta \gamma_{1,(n)}/\gamma_{2,(n)}=\theta \frac{\alpha {\left\|b^{\left(n\right)}-u^{\left(n\right)}\right\|}_{2}^{2}+\beta \langle \lambda Kb^{\left(n\right)}-v^{\left(n\right)},\lambda K{\hat{y}}^{\left(n\right)}-v^{\left(n\right)}\rangle }{{\left\|d_{1}{}^{\left(n\right)}\right\|}_{2}^{2}+{\left\|d_{2}{}^{\left(n\right)}\right\|}_{2}^{2}}.$$

where θ is a nonnegative scaling factor, and

$$d_{1}{}^{\left(n\right)}=\alpha (b^{\left(n\right)}-u^{\left(n\right)})+\beta \lambda^{T}K^{T}(\lambda K{\hat{y}}^{\left(n\right)}-v^{\left(n\right)}),\;$$

$$d_{2}{}^{\left(n\right)}=v^{\left(n\right)}-\lambda Ku^{\left(n\right)}.$$

In conclusion, the proposed method is summarized in Algorithm 1.
**Algorithm 1** Iterative Proximal Adaptive Descent Algorithm (IPAD)**Input:** choose α>β>0,t∈R,θ∈(0,2) such that the constant              $c=1-t\sqrt{\beta }{\left\|\lambda K\right\|}_{2}/2\sqrt{\alpha }\geq 0.$ **Initialization: **
$u^{0},b^{0},y^{0}$ **While **
$n\leq n_{\max}$$\;\mathrm{or}\;{\left\|b^{\left(n\right)}-b^{\left(n-1\right)}\right\|}_{2}/{\left\|b^{\left(n-1\right)}\right\|}_{2}\leq \varepsilon$, $1.\;(Proximal\;step).\;\mathrm{Compute}\;(u^{\left(n\right)},{\hat{y}}^{\left(n\right)},v^{\left(n\right)})$ via (8)–(11). $2.\;(Descent\;step).\;\mathrm{Compute}\;(b^{\left(n+1\right)},y^{\left(n+1\right)})$ via (12)–(13). Set n=n+1. **End while** **Output:**$\;b^{\left(n\right)}$

**Remark** **1.** *The constant c satisfies c≥0 in Algorithm 1 and is the constraint of the parameters and operator; its derivation is demonstrated in the convergence analysis subsection in detail.*

### 2.3. Convergence Analysis

This subsection establishes the global convergence of the proposed Algorithm 1. Note that global convergence means that the generated iterative sequence converges to a critical point, which follows the usage of global convergence for the extended splitting method in monotone inclusion problems [35]. Meanwhile, in order to facilitate the derivation process, the regularization parameter λ is incorporated into the operator K, i.e., K:=λK. According to the update of variables $\left(u^{\left(n\right)},v^{\left(n\right)}\right)$, they satisfy the following conditions:

(15) $$\{\begin{array}{c} \alpha b^{\left(n\right)}-K^{T}y^{\left(n\right)}-\alpha u^{\left(n\right)}\in \partial G(u^{\left(n\right)})\\ y^{\left(n\right)}+\beta K{\hat{y}}^{\left(n\right)}-\beta v^{\left(n\right)}\in \partial b(v^{\left(n\right)}) \end{array}.$$

Then, we introduce the necessary lemma 1 for deriving convergence theorem 1, whose proofs are given in Appendix B and Appendix C.

**Lemma** **1.** *Let* $\left\{(b^{\left(n\right)},y^{\left(n\right)})\right\}$ *be the sequence generated by Algorithm 1, the following inequality holds:*

$$\begin{array}{l} \langle b^{\left(n\right)}-b^{*},d_{1}{}^{\left(n\right)}\rangle +\langle y^{\left(n\right)}-y^{*},d_{2}{}^{\left(n\right)}\rangle \\ \geq (1-\frac{t\sqrt{\beta }{\left\|K\right\|}_{2}}{2\sqrt{\alpha }})(\alpha {\left\|b^{\left(n\right)}-u^{\left(n\right)}\right\|}_{2}^{2}+\beta {\left\|Kb^{\left(n\right)}-v^{\left(n\right)}\right\|}_{2}^{2}), \end{array}$$

*where* $\left(b^{*},y^{*}\right)$ *is the critical point of the objective function in problem (5).*

**Theorem** **1.** *Let* $\left\{(b^{\left(n\right)},y^{\left(n\right)})\right\}$ *be the sequence generated by Algorithm 1. For any given *t>0,α>β>0, *if *$1-\frac{t\sqrt{\beta }{\left\|K\right\|}_{2}}{2\sqrt{\alpha }}\geq 0$ *and* 0<θ<2*, then the sequence globally converges to a critical point *$\left(b^{*},y^{*}\right)$ *of the objective function.*

## 3. Results

In this section, a simulated phantom and a more realistic thorax dataset are used to assess the performance of the proposed method. The comparison methods chosen are the filtered back projection (FBP) reconstruction with a subsequent direct matrix inversion (Directdecom), the three-material ESART algorithm with OS acceleration (OSesart), and the nonconvex ADMM (NcADMM) method. To further clarify the effectiveness of the proposed IPAD algorithm, the root mean square error (RMSE), the peak signal-to-noise ratio (PSNR), and the structural similarity index (SSIM) [36] are employed for quantitative assessment. In addition, the initial guess is chosen as zero for iterative methods and the OS number is set to 90. The number of iterations for the OSesart and IPAD methods are set to 100 for both simulation data and thorax data. The number of iterations for the NcADMM method are set to 1000 and 100 for simulation data and thorax data, respectively. All the methods in our experiments are implemented on the workstation equipped with an Intel(R) Xeon(R) Gold 6234 @3.30GHz CPU and NVIDIA Quadro RTX 6000 GPU.

### 3.1. Algorithm Investigation

**Figure 1:**
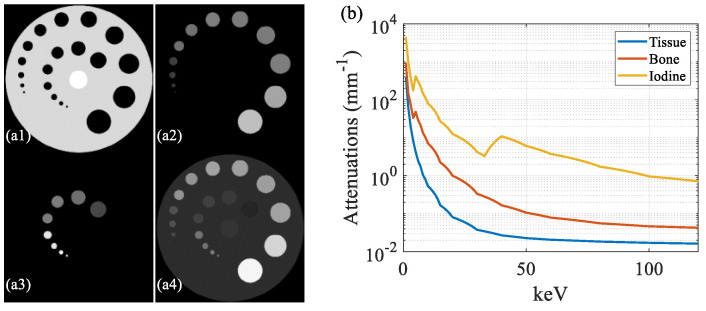

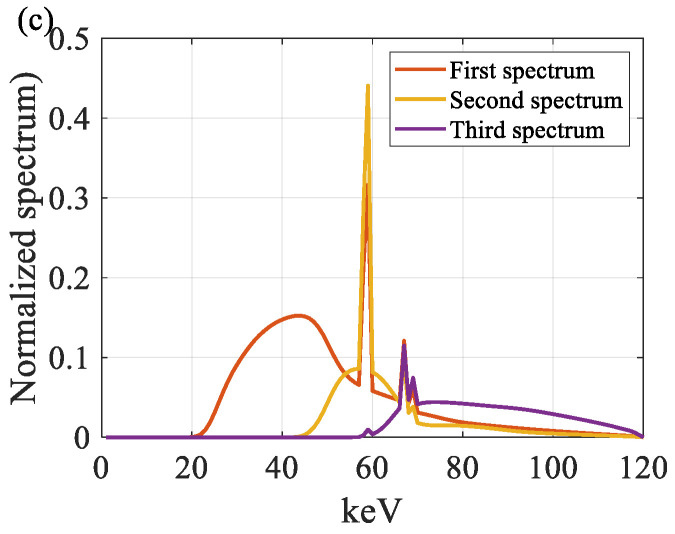
(**a1**–**a4**) Simulation phantom that consists of (**a1**) tissue, (**a2**) bone, and (**a3**) iodine, and (**a4**) represents the simulated object. (**b**) Attenuations of different materials. (**c**) Three normalized spectrums were used in the simulation experiments.

The simulated phantom with 256 × 256 pixels is composed of three basis materials, i.e., tissue, bone, and iodine, as shown in Figure 1(a1–a4), where the bone contains 12 circles, increased from a radius of 0.2 mm to 2.4 mm with an increment of 0.2 mm. Iodine consists of eight circles with a radius increased from 0.2 mm to 1.6 mm and the concentration of the corresponding circles decreases from 12 mg/mL to 5 mg/mL. The attenuation curves of three materials are shown in Figure 1b, which are provided by the National Institute of Standards and Technology (NIST). The distances from the source to the object and detector are 300.0 and 600.0 mm, respectively. Projections are obtained through 360 views on average distributed in 360° via fan beam scanning, and the number of detector units is 512 with a size of 0.124 mm. The source spectrum is simulated at 120 kVp through SpekCalc software [37] with 1.2 mm aluminum filtration and the three energy thresholds are set to 25, 51, and 66 keV. To make the simulation more realistic, the three spectra (shown in Figure 1c) are obtained by combing the energy bins with the detector response model utilized in reference [14].

#### 3.1.1. Noise-Free Simulation

**Figure 2:**
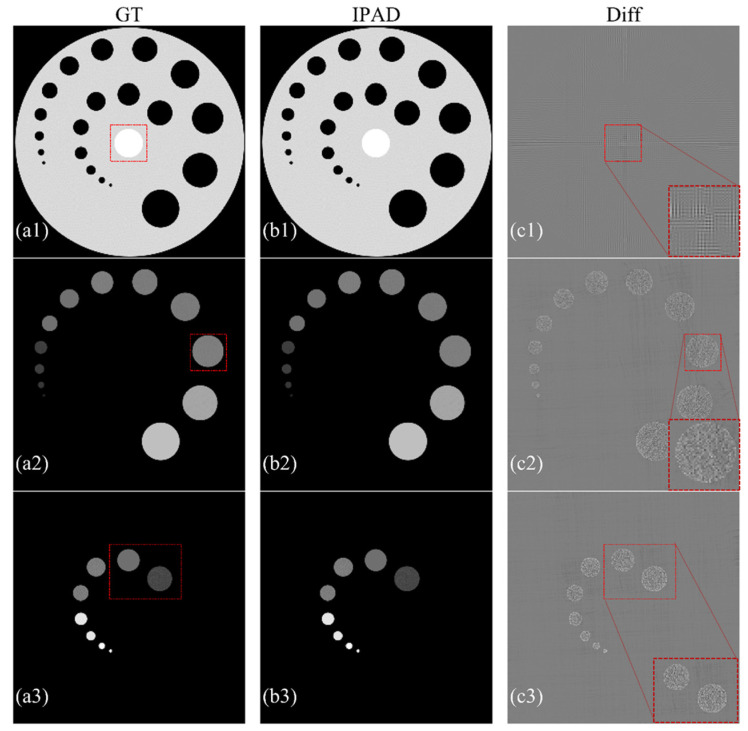
Results of the noise-free simulation dataset obtained by the proposed IPAD method. (**a1**–**a3**), (**b1**–**b3**), and (**c1**–**c3**) represent the tissue, bone, and iodine obtained by the GT, the proposed IPAD method, and their differential images, respectively, where the display windows of the first two columns are [0.01 1], [0.01 1], and [0.01 0.5], respectively. The corresponding differential maps are shown in a 0.1% grayscale window [−0.005 0.005] for tissue and a 0.01% grayscale window [−0.0001 0.0001] for bone and iodine.

**Figure 3:**
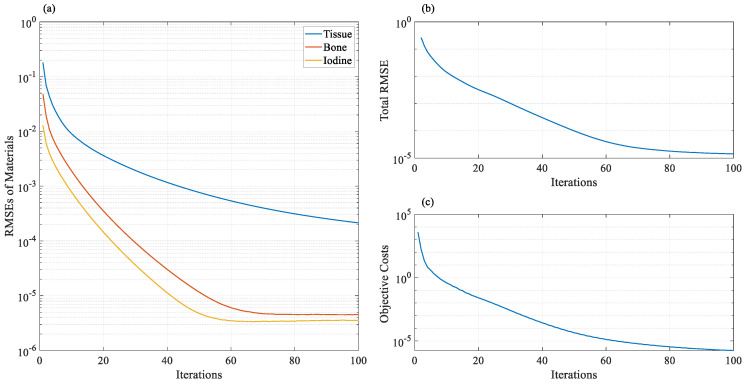
Convergence behaviors of the proposed method, where (**a**) indicates the RMSEs of three basis materials, (**b**) indicates the total RMSE of the proposed IPAD method, and (**c**) represents the costs of the objective function.

First, the ideal, noise-free simulated data are applied to verify the accuracy of the proposed IPAD method. Figure 2 shows the images of the proposed method, where columns (a) to (c) indicate the ground truth (GT), the IPAD method, and their differential images, respectively. According to the results shown in Figure 2, the material maps reconstructed by the IPAD method are close to the given phantom maps in most areas. The differential images (Diff) of tissue, bone, and iodine are displayed on a grayscale of 0.1%, 0.01%, and 0.01% in Figure 2, respectively. Further, the regions of interest (ROIs) marked by the red box in the last column of Figure 2 further indicate the effectiveness of the proposed method. In addition, the convergence performance of the IPAD method is shown in Figure 3, where (a) represents the RMSE reduction curves of the three basis materials with the increasing iteration number. It can be seen that the orders of magnitude that these materials can achieve are 1 × 10^−4^, 1 × 10^−6^, and 1 × 10^−6^, respectively. Further, (b) indicates that the total RMSE of the IPAD method has reached about 1 × 10^−5^ at 100 iterations. In addition to the RMSE curves, the third figure in Figure 3 further plots the convergence behavior of the objective function in the reconstructed model and its downward trend also indicates that the algorithm can continuously converge with the increase in iterations.

**Figure 4:**
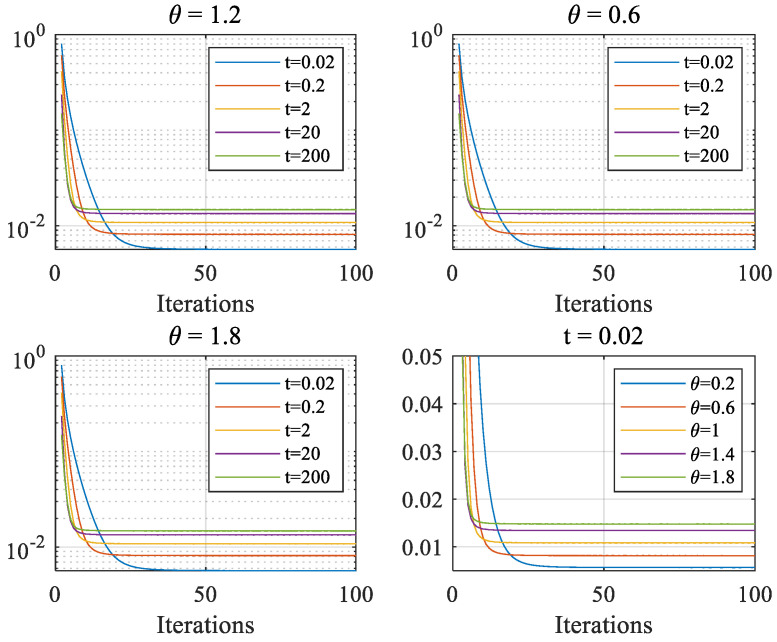
Convergence metrics of RMSEs of different values of t,θ.

Furthermore, there are two factors t,θ that affect the convergence of the proposed IPAD algorithm, where t is designed to balance the current point and the former point, and θ is used to modify the adaptive descent step. According to the convergence analysis of the algorithm, the value of t is chosen from the set {0.02,0.2,2,20,200}, and θ is selected from the set {0.2,0.4,0.6,0.8,1,1.2,1.4,1.6,1.8}. Finally, the values of t and θ are set to 0.02 and 0.2 according to the RMSEs convergence behaviors plotted in Figure 4, respectively.

#### 3.1.2. Performance under Different Noise Levels

**Figure 5:**
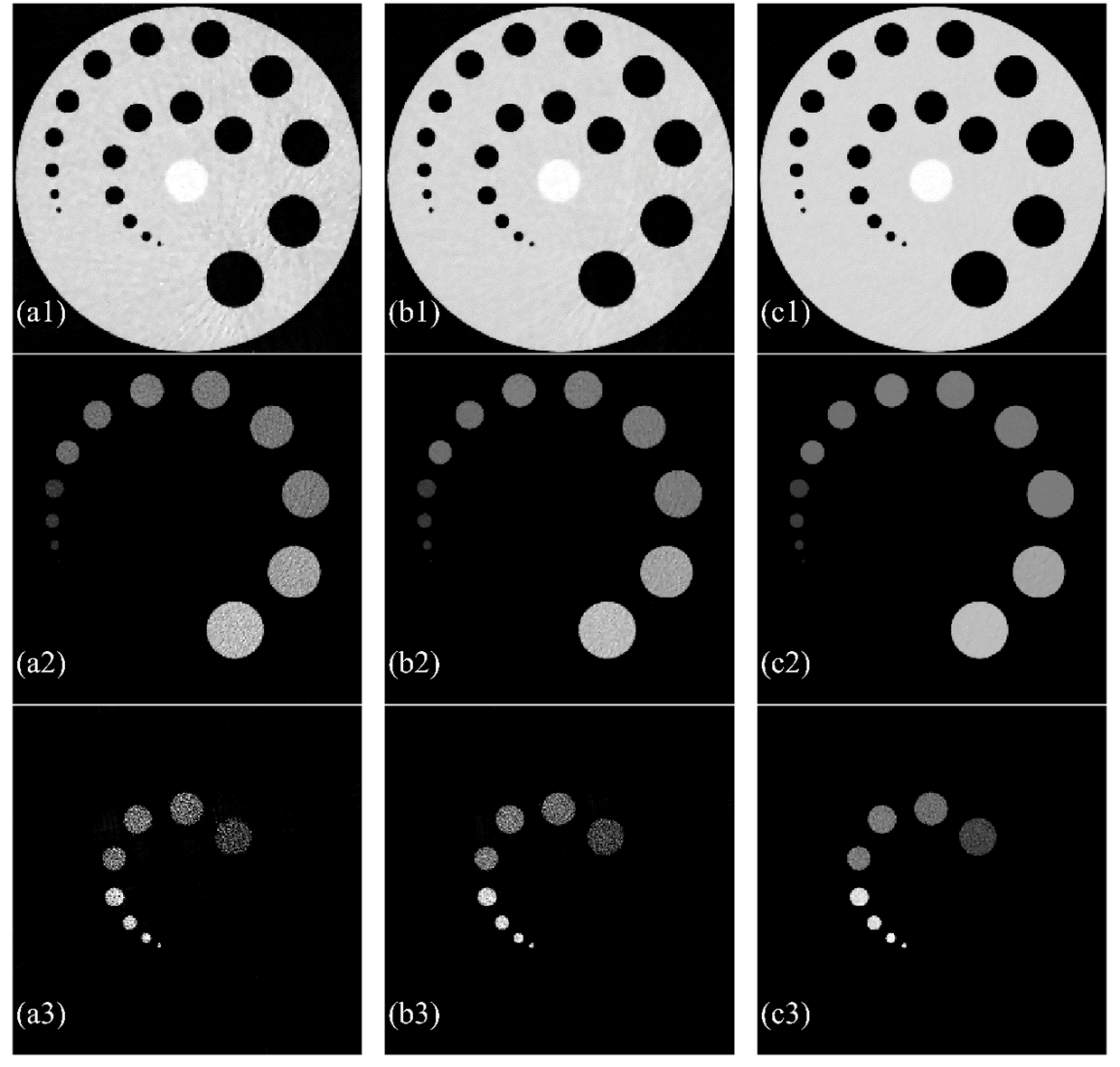
Three material reconstruction results of the proposed IPAD method at different noise levels. (**a1**–**a3**), (**b1**–**b3**), and (**c1**–**c3**) represent the tissue, bone, and iodine ob-tained by the proposed IPAD method at the noise I_0_ = 5 × 10^5^, 1 × 10^6^, 5 × 10^6^, respectively. The display windows of the three materials are [0.01 1], [0.01 1], and [0.01 0.5], respectively.

To illustrate the robustness of the proposed IPAD method, different Poisson noise levels are considered to be added to the simulation datasets. In this work, Poisson noise is generated and injected into the projections to simulate noisy measurements as

(16) $$P=\frac{I_{0}{}^{k}}{k!}e^{-I_{0}},p_{i}=P\cdot \exp(-p_{0}),$$

where $I_{0}$ stands for the number of incident X-ray photons, and $p_{0},p$ are the measured projection data and the photons of adding noise collected by the detector unit i, respectively. k is the index of the detector unit. Different noise levels of I_0_ = 5 × 10^5^, 1 × 10^6^, 5 × 10^6^ are considered to validate the performance of the proposed algorithm. The corresponding results of material reconstruction are shown in Figure 5. Additionally, the regularization parameters $\lambda =[\lambda_{1},\lambda_{2},\lambda_{3}]$ are also adjusted according to the different noise levels. In other words, the regularization parameters of the three basis materials are also different. Empirically, the parameters $\lambda_{1}$ and $\lambda_{2}$ of water and bone materials are selected from the same set $\left\{10^{-7},10^{-6},10^{-5},10^{-4},10^{-3}\right\}$, and the parameter $\lambda_{3}$ of iodine material is selected from the set $\left\{10^{-6},10^{-5},10^{-4},10^{-3},10^{-2}\right\}$. The relatively optimized values of the three parameters are fixed at $10^{-6},10^{-6},10^{-5}$ by enough trials. For different noise levels, the proposed IPAD algorithm can also have a better performance in suppressing noise.

### 3.2. Comparison Experiments

In this subsection, comparisons with the state-of-art algorithms will be carried out to further verify the performance of the proposed method. To make the data more realistic, Poisson noise is added to the obtained projections to simulate image noises. In this work, we set I_0_ = 1 × 10^6^ and I_0_ = 1 × 10^7^ to validate the effectiveness of the proposed algorithm.

**Figure 6:**
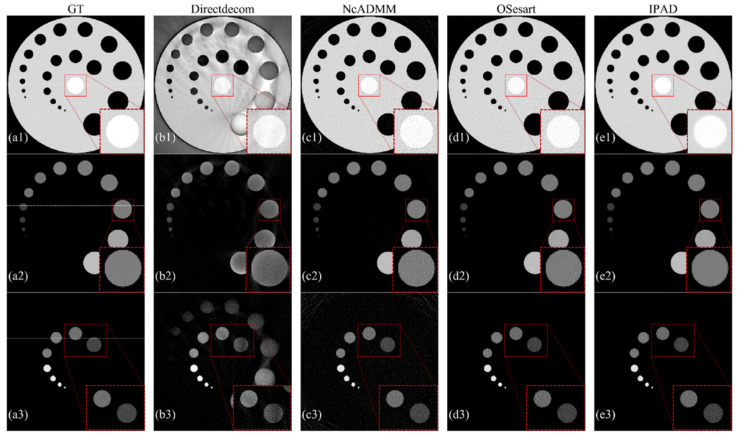
Results of the simulation dataset obtained by different methods with added noise (I_0_ = 1 × 10^7^). (**a1**–**a3**), (**b1**–**b3**), (**c1**–**c3**), (**d1**–**d3**), and (**e1**–**e3**) represent the tissue, bone, and iodine obtained by the GT, Directdecom, NcADMM, OSesart, and the proposed IPAD method, and their corresponding enlarged images, respectively. The display windows of the three materials are [0.01 1], [0.01 1], and [0.01 0.5], respectively.

**Figure 7:**
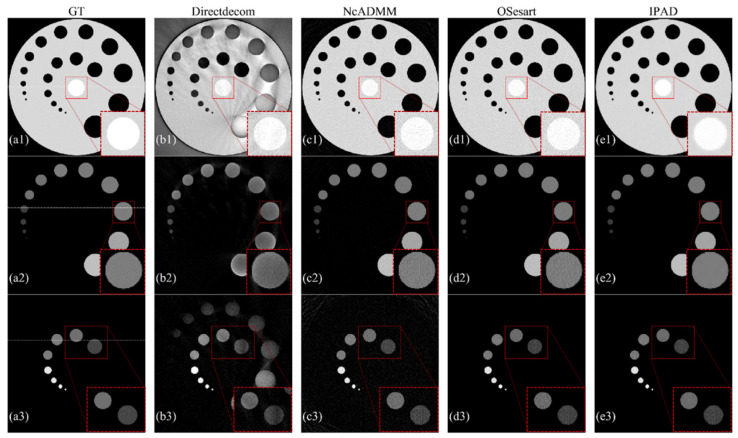
Results of simulation dataset obtained by different methods with adding noise (I_0_ = 1 × 10^6^). (**a1**–**a3**), (**b1**–**b3**), (**c1**–**c3**), (**d1**–**d3**), and (**e1**–**e3**) represent the tissue, bone, and iodine obtained by the GT, Directdecom, NcADMM, OSesart, and the proposed IPAD method, and their corresponding enlarged images, respectively. The display windows of the three materials are [0.01 1], [0.01 1], and [0.01 0.5], respectively.

Figure 6 and Figure 7 show the reconstruction results of different methods at two different noise levels, where columns (a) to (e) represent the images of GT, Directdecom, OSesart, NcADMM, and the proposed IPAD method, respectively. Compared to GT, the results of the Directdecom method are relatively inaccurate, especially in the images of iodine, where parts of the bone are misclassified as iodine. From the enlarged areas marked by the red box in Figure 6(b1–b3) and Figure 7(b1–b3) of the Directdecom method, it is further confirmed that noise increases the difficulty of direct decomposition.

The NcADMM method has the ability to obtain relatively fine structures of different materials. However, obvious noise still can be observed on the maps of iodine material in Figure 6(c3) and Figure 7(c3). The OSesart and the proposed IPAD method have better performance than the results of Directdecom and NcADMM methods. However, as shown in the magnified regions of Figure 6(d1,d2) and Figure 7(d1,d2), the noises remain in the reconstruction results of OSesart due to its incapacity to denoise. Compared with the above methods, the proposed IPAD algorithm has advantages in denoising while maintaining the accuracy of the reconstructed basis materials, especially in the ROIs shown in Figure 6(e2) and Figure 7(e2).

Quantitative evaluations are listed in Table 1. Taking the noise level I_0_ = 1 × 10^6^ as an example to illustrate the overall performance of different methods, it can be seen from Table 1 that the averaged PSNR for three materials of the proposed IPAD algorithm is up to 42.555 dB, which increased PSNRs by 22.546 dB, 14.149 dB, and 4.072 dB compared with those of the Directdecom, NcADMM, and OSesart methods, respectively. Further, the IPAD method obtains the highest SSIM index of 0.908 while the SSIMs of other methods are below 0.9. In addition, the RMSE of the proposed IPAD algorithm converged to 0.0093, which reduced the RMSEs by 92.79%, 76.21%, and 40.76% compared to those of the Directdecom, NcADMM, and OSesart methods. The computation costs of three iterative algorithms are listed in Table 2. It is evident that the proposed method outperforms NcADMM in terms of computational efficiency, while the former is comparable to that of OSesart.

**Figure 8:**
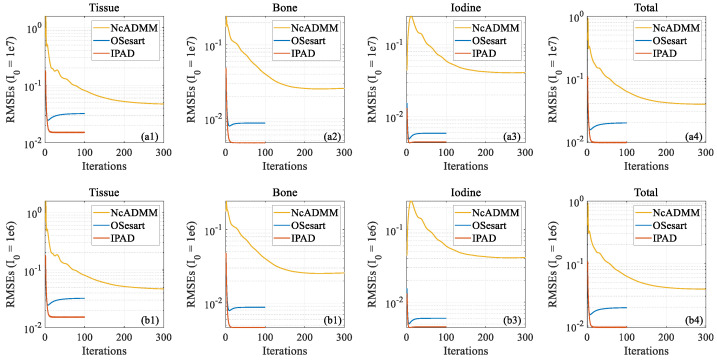
Convergence behaviors of the three iterative algorithms under different noise levels, where the first row represents the RMSEs of noisy data (I_0_ = 1 × 10^7^), and the second row represents the RMSEs of noisy data (I_0_ = 1 × 10^6^). (**a1**–**a4**), and (**b1**–**b4**) represent the RMSEs of tissue, bone, iodine, and total of different methods at two noise levels.

**Figure 9:**
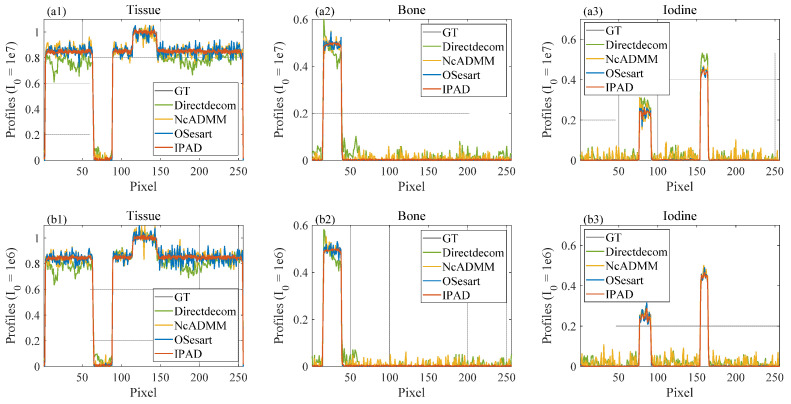
Profiles of different methods at different noise levels. (**a1**–**a3**), and (**b1**–**b3**) represent the profiles of tissue, bone, and iodine of different methods at two noise levels.

Figure 8 shows the convergence behaviors of the three iterative methods at different noise levels. The first three columns denote the RMSEs of three different basis materials, and the last column indicates the total RMSEs of the three iterative methods. The OSesart and the proposed IPAD methods still keep a rapid downward trend within the first 20 iterations, while the results of the OSesart method come down a little bit slower; the former is then greatly affected by adding noise in subsequent iterations. It can be seen that the RMSE curves of the OSesart method in Figure 8 have a rising state, which is because the OSesart method lacks the ability to denoise, and noise leads to an increasing difference between the reconstruction results and the true value. Over the total of 100 iterations, the proposed IPAD method has been found to be convergently stable. As for the NcADMM method, it is also convergent under 1000 iterations, but its decreasing speed is the slowest compared with the other two iterative methods, and the final reconstruction accuracy does not improve with the increase in iterations when shown in the first 300 iterations. Therefore, it can be observed in the total RMSE curves in Figure 8(a4,b4) that the proposed IPAD method is relatively stable, while the OSesart method is prone to convergence under the influence of noise, and the NcADMM method has disadvantages in descending accuracy and speed. The line profiles, drawn from pixels along the white dashed line in Figure 5(a1) and Figure 6(a1), are further plotted in Figure 9. This demonstrates that the proposed IPAD method obtains more accurate structures and details than the other compared methods.

### 3.3. Thorax Dataset Verification

The thorax dataset (https://www.ircad.fr/research/data-sets/respiratory-cycle-3d-ircadb-02/, accessed on 1 July 2018); downloaded to verify the performance of the algorithm in clinical experiments (The dataset is publicly available, and no ethical statements are involved). The public dataset contains 167 slices in total. We extract the 160th slice to implement our method with a more complex phantom. The selected slice is segmented into three components by the labels given in the dataset, i.e., tissue, bone, and lungs, where the lungs are supposedly injected with iodine and the concentration of iodine is set at 15 mg/mL. The corresponding attenuation coefficients of the three basis materials are obtained by NIST. Then, the projections are determined by the forward transmission model (4) with three energy bins. Similar to the simulation, the spectrum is derived from a tube voltage of 120 kVp and a 12 mm aluminum filter, and the three energy windows are separated by the thresholds 30, 56, and 70 keV, respectively. The distances of the source to the object and source to detector are 1000.0 mm and 1500.0 mm, respectively. The reconstructed image pixel is 512 × 512 with each pixel size of 0.961 mm, and the number of photon-counting detector units is 1024 with each size of 0.7208 mm. Projection views are also set to 360 in the 360° range. Similarly, it is assumed that the measured projection data are contaminated by Poisson noise with level I_0_ = 1 × 10^6^.

**Figure 10:**
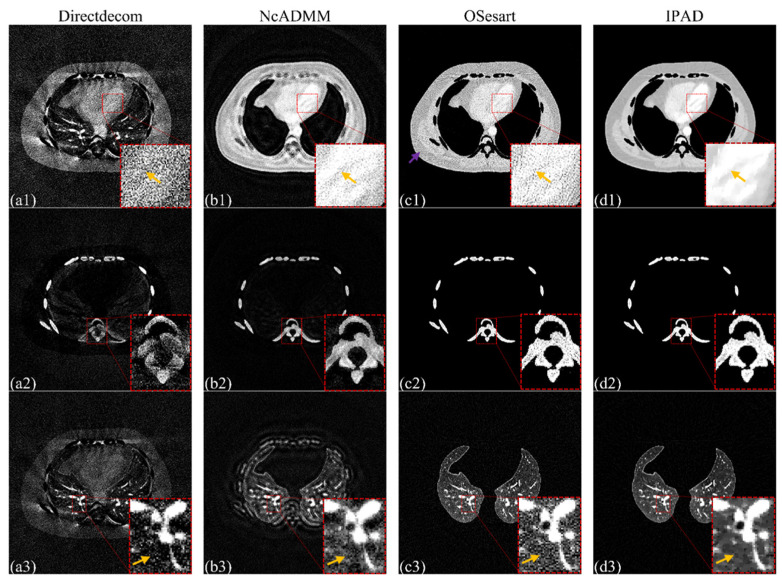
Results of the thorax dataset obtained by different methods with added noise (I_0_ = 1 × 10^6^). (**a1**–**a3**), (**b1**–**b3**), (**c1**–**c3**), and (**d1**–**d3**) represent the tissue, bone, and iodine obtained by Directdecom, NcADMM, OSesart, and the proposed IPAD. And the corresponding display windows are [0.01 0.8], [0.01 0.65], and [0.01 0.6], respectively.

Figure 10 shows the results of the reconstructed thorax data by different methods, where columns (a) to (e) represent the reconstructed maps of the Directdecom, NcADMM, OSesart, and the proposed IPAD algorithms, respectively. The rows from top to bottom indicate the three basis materials: tissue, bone, and lungs. As shown in Figure 10(a1,a3), the tissue and parts of the lungs are indistinguishably obtained by the Directdecom method. The results of NcADMM show that its tissue map is relatively accurate, while it contains some noise on the bone map and some bones are misclassified on the lung map. Furthermore, there are obvious streak-like artifacts in the tissue image reconstructed by OSesart, as marked in the purple arrow in Figure 10(c1). Compared with the abovementioned two methods, the proposed IPAD algorithm has better performance in material decomposition accuracy, artifacts, and noise suppression. Furthermore, as indicated by the yellow arrows in the first and third rows of magnified areas, the IPAD method reconstructs a clear texture, while the other two methods contain a large amount of noise and the textures are not so clear.

**Figure 11:**
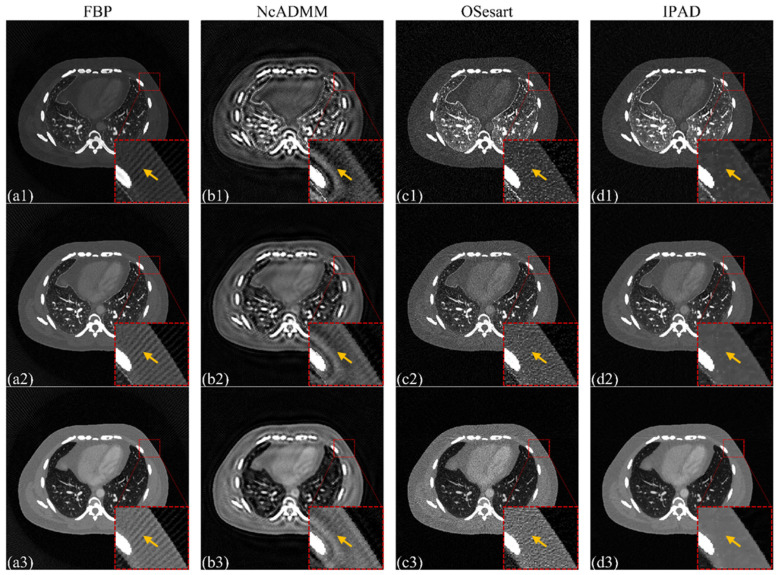
Virtual monochromatic images of thorax dataset at energy 40, 65, and 90 keV. (**a1**–**a3**), (**b1**–**b3**), (**c1**–**c3**), and (**d1**–**d3**) represent the virtual monochromatic images of FBP, NcADMM, OSesart, and the proposed IPAD method, respectively. Note that the “FBP” means there are the results reconstructed by FBP in three energy bins. The corresponding display windows are [0 0.08], [0 0.04], and [0 0.025], respectively.

To further illustrate the effectiveness of the one-step method in eliminating beam-hardening artifacts, virtual monochromatic images are shown in Figure 11. Notice that the first column in Figure 11 represents the reconstructions of FBP through three energy bins. The last three columns of Figure 11 display the results of virtual monochromatic images between the three iterative methods at single energy 40, 65, and 90 keV, respectively. Compared to the results reconstructed by FBP, the three iterative one-step methods greatly eliminate the influence of beam-hardening artifacts, as can be observed from the ROIs marked by the yellow arrows in Figure 11. However, the NcADMM method still has ambiguous structures due to noise. In addition, it is obvious that the proposed IPAD algorithm has advantages in noise suppression compared to the OSesart method.

## 4. Discussion

In this paper, we consider that the ill-condition of the multi-material reconstruction problem increases with the increase in the number of materials, especially in the case that each energy spectrum has a consistent scanning path. It is more difficult to obtain accurate distributions of basis materials based on one-step methods. Aiming at this situation, a new iterative one-step multi-material reconstruction method is developed, in which an adaptive proximal descent step is designed to constantly modify the direction of the algorithm during the update process. The proposed method further combines sparsity-based TV regularization to integrate the noise suppression in each iteration of material reconstruction, which enables us to obtain a more stable solution. Moreover, convergence analysis under some mathematical assumptions is derived that the proposed algorithm is guaranteed by theory according to its update scheme. In addition, several numerical experiments are carried out to verify the effectiveness of the proposed IPAD method. The results show that the practical performance is consistent with the original design, and it can obtain a relatively stable solution while suppressing noise. The monochromatic image results, as shown in Figure 11, further indicate the proposed method has the ability to eliminate beam-hardening artifacts.

Although the proposed algorithm proves that it is convergent in theory, model-driven methods based on certain assumptions cannot fully express the physical mechanism for the realistic application of CT imaging; for example, the response of detector units is different for certain spectrum, and noise in the measured projections is easily multiple amplified in the reconstructed process of basis materials. Noise disturbance is a huge instability factor for the convergence of the algorithm. As a result, data-driven methods for material reconstruction have also been developed, such as a butterfly network by Zhang et al. [38] to realize material decomposition based on an image domain under dual energy. Fang et al. applied the unsupervised denoising method called Noise2Noise [39] as prior knowledge to estimate the material maps directly from the raw projection data [40]. In addition, other researchers have also found the deep learning-based method has certain advantages in spectral CT imaging [41,42]. These methods also encourage us to combine model-driven and data-driven methods to achieve accurate decomposition of materials by eliminating the influence of beam-hardening artifacts while suppressing noise in the future.

## 5. Conclusions

This paper proposes a one-step basis material reconstruction algorithm based on proximal function. First, the reconstruction model consists of a data fidelity function and a TV regularization term. Then, we design an iterative proximal adaptive descent algorithm to solve this optimization model. Moreover, the convergence analysis is established to support that the iterative sequence generated by the proposed algorithm converges to a critical point of the original optimization model. Furthermore, an ordered subset technique is applied to accelerate the algorithm. The simulation and thorax experiments verify the effectiveness of the proposed method in basis material reconstruction and also the capabilities in suppressing beam hardening and Poisson noise.

## Figures and Tables

**Table 1:** Quantitative results of different methods.

		Noisy (I_0_ = 1 × 10^7^)			Noisy (I_0_ = 1 × 10^6^)		
Algorithm	Materials	PSNR	SSIM	RMSE	PSNR	SSIM	RMSE
Directdecom	Tissue	11.970	0.454	0.252	11.626	0.443	0.262
	Bone	23.009	0.269	7.07 × 10^−2^	23.582	0.320	6.62 × 10^−2^
	Iodine	23.620	0.326	6.59 × 10^−2^	24.818	0.371	5.74 × 10^−2^
	Averaged	19.533	0.350	0.130	20.009	0.378	0.129
NcADMM	Tissue	26.609	0.505	4.67 × 10^−2^	26.611	0.505	4.67 × 10^−2^
	Bone	31.546	0.618	2.65 × 10^−2^	31.472	0.614	2.67 × 10^−2^
	Iodine	27.213	0.357	4.36 × 10^−2^	27.134	0.353	4.4 × 10^−2^
	Averaged	28.456	0.493	3.89 × 10^−2^	28.406	0.491	3.91 × 10^−2^
OSesart	Tissue	29.836	0.669	3.22 × 10^−2^	29.819	0.668	3.23 × 10^−2^
	Bone	41.236	0.968	8.72 × 10^−3^	41.176	0.967	8.74 × 10^−3^
	Iodine	44.609	0.983	5.91 × 10^−3^	44.455	0.983	6.02 × 10^−3^
	Averaged	38.560	0.873	1.56 × 10^−2^	38.483	0.873	1.57 × 10^−2^
IPAD	Tissue	34.846	0.787	1.81 × 10^−2^	34.818	0.787	1.82 × 10^−2^
	Bone	45.287	0.951	5.41 × 10^−3^	45.420	0.950	5.42 × 10^−3^
	Iodine	47.643	0.986	4.11 × 10^−3^	47.426	0.987	4.31 × 10^−3^
	Averaged	42.592	0.908	9.2 × 10^−3^	42.555	0.908	9.3 × 10^−3^

**Table 2:** Computation costs of three iterative algorithms for one iteration.

Computation Costs	NcADMM	OSesart	IPAD
Time (unit: second)	64.898	24.451	25.520

## Data Availability

The data and source code are available from the corresponding author upon reasonable request upon the publication of this work.

## Appendix A. Solving Scheme for Proximal Step

We will describe the details of the solving scheme for the proximal step in Algorithm 1. First, rewrite the first-order Taylor expansion of the forward projection model (4) at the current point $u^{\left(n\right)}$ for the s-th spectrum and omit the high-order remainder; the non-linear measurements can be expressed as

(A1) $$p_{sj}(u)=p_{sj}(u^{\left(n\right)})+\sum_{k=1}^{K}\frac{\Phi_{s,k,j}^{\left(n\right)}}{q_{s,j}^{\left(n\right)}}e_{j,k}^{\left(n\right)},$$

where

$$\begin{array}{l} p_{sj}(u^{\left(n\right)})=-\ln\sum_{m=1}^{M}S_{sjm}\exp(-\sum_{k=1}^{K}\mu_{mk}{\left[Au_{k}^{\left(n\right)}\right]}_{j}),\\ q_{s,j}^{\left(n\right)}=\sum_{m=1}^{M}S_{sjm}\exp(-\sum_{k=1}^{K}\mu_{mk}{\left[Au_{k}^{\left(n\right)}\right]}_{j}),\\ \Phi_{s,k,j}^{\left(n\right)}=\sum_{m=1}^{M}S_{sjm}\cdot \mu_{mk}\cdot \exp(-\sum_{k=1}^{K}\mu_{mk}{\left[Au_{k}^{\left(n\right)}\right]}_{j}),\\ e_{j,k}^{\left(n\right)}={\left[A(u_{k}-u_{k}^{\left(n\right)})\right]}_{j}. \end{array}$$

Therefore, the errors $e_{j,k}^{\left(n\right)}(k=1,\ldots ,K)$ can be computed directly by rewriting the Equation (A1) at the current ray j, i.e.,

(A2) $$\left(\begin{array}{c} e_{j,1}^{\left(n\right)}\\ \cdot \cdot \cdot \\ e_{j,K}^{\left(n\right)} \end{array}\right)={\left(\begin{array}{ccc} \frac{\Phi_{1,1,j}^{\left(n\right)}}{q_{1,j}^{\left(n\right)}} & \cdot \cdot \cdot & \frac{\Phi_{1,K,j}^{\left(n\right)}}{q_{1,j}^{\left(n\right)}}\\ \cdot \cdot \cdot & & \\ \frac{\Phi_{S,1,j}^{\left(n\right)}}{q_{S,j}^{\left(n\right)}} & \cdot \cdot \cdot & \frac{\Phi_{S,K,j}^{\left(n\right)}}{q_{S,j}^{\left(n\right)}} \end{array}\right)}^{-1}\left(\begin{array}{c} p_{1j}^{M}-p_{1j}(u^{\left(n\right)})\\ \cdot \cdot \cdot \\ p_{Sj}^{M}-p_{Sj}(u^{\left(n\right)}) \end{array}\right),l\in \Omega_{s},s=1,\ldots ,S.$$

Then, the OS technology further divides the rays $j\in \Omega_{s}$ into L subsets $\left\{\Omega_{s_{1}},\ldots ,\Omega_{s_{L}}\right\}$ and updates the residuals indexed by one of the subsets each time in this paper. Finally, these residuals in one of the subsets are applied to update the next new estimates $u_{k}^{\left(n+1\right)}(k=1,\ldots ,K)$ via the following formula:

(A3) $$u_{k}^{\left(n+1\right)}={\tilde{u}}_{k}^{\left(n\right)}+\frac{\eta }{\sum_{j\in \Omega_{s_{l}}}A_{j}}\sum_{j\in \Omega_{s_{l}}}A_{j}\left(\frac{e_{j,k}^{\left(n\right)}}{\sum_{i=0}^{I-1}a_{j,n}}\right),(k=1,2,\ldots ,K),$$

where ${\tilde{u}}_{k}^{\left(n\right)}=\alpha (u^{\left(n\right)}-b^{\left(n\right)})+\lambda^{T}K^{T}y^{\left(n\right)}$ is the first-order derivation with respect to u of the second term in the proximal step. $A_{j}=[a_{j1},a_{j2},\ldots ,a_{jI}]$ represents the contributions of each pixel to the projection along the j-th X-ray path.

## Appendix B. Proof of Lemma 1

**Proof.** The function G(b) is convex with the linearization process by the first-order Taylor expansion. Combing the fact that the sub-differential of any closed proper convex function in an infinite dimensional space is maximal monotone [43], thus, we use the monotonicity of ∂G together with (15) to obtain

$$\langle u^{\left(n\right)}-b^{*},\alpha b^{\left(n\right)}-K^{T}y^{\left(n\right)}-\alpha u^{\left(n\right)}+K^{T}y^{*}\rangle \geq 0.$$

Further, let $u^{\left(n\right)}-b^{*}=b^{\left(n\right)}-b^{*}-(b^{\left(n\right)}-u^{\left(n\right)})$, then

(A4) $$\langle b^{\left(n\right)}-b^{*},\alpha b^{\left(n\right)}-\alpha u^{\left(n\right)}\rangle -\langle Ku^{\left(n\right)}-Kb^{*},y^{\left(n\right)}-y^{*}\rangle \geq \alpha {\left\|b^{\left(n\right)}-u^{\left(n\right)}\right\|}_{2}^{2}.$$

Meanwhile, using the monotonicity of ∂B, we obtain

$$\langle v^{\left(n\right)}-Kb^{*},y^{\left(n\right)}+\beta K{\hat{y}}^{\left(n\right)}-\beta v^{\left(n\right)}-y^{*}\rangle \geq 0.$$

Together with $v^{\left(n\right)}-Kb^{*}=Kb^{\left(n\right)}-Kb^{*}-(Kb^{\left(n\right)}-v^{\left(n\right)})$, it implies

(A5) $$\begin{array}{l} \langle b^{\left(n\right)}-b^{*},\beta K^{T}K{\hat{y}}^{\left(n\right)}-\beta K^{T}v^{\left(n\right)}\rangle +\langle v^{\left(n\right)}-Kb^{*},y^{\left(n\right)}-y^{*}\rangle \\ \geq \langle Kb^{\left(n\right)}-v^{\left(n\right)},\beta K{\hat{y}}^{\left(n\right)}-\beta v^{\left(n\right)}\rangle . \end{array}$$

Summing up Inequalities (A4) and (A5), we have

(A6) $$\begin{array}{l} \langle b^{\left(n\right)}-b^{*},d_{1}{}^{\left(n\right)}\rangle +\langle y^{\left(n\right)}-y^{*},d_{2}{}^{\left(n\right)}\rangle \\ \geq \alpha {\left\|b^{\left(n\right)}-u^{\left(n\right)}\right\|}_{2}^{2}+\langle Kb^{\left(n\right)}-v^{\left(n\right)},\beta K{\hat{y}}^{\left(n\right)}-\beta v^{\left(n\right)}\rangle \\ =\alpha {\left\|b^{\left(n\right)}-u^{\left(n\right)}\right\|}_{2}^{2}+\beta {\left\|Kb^{\left(n\right)}-v^{\left(n\right)}\right\|}_{2}^{2}-t\beta \langle Kb^{\left(n\right)}-v^{\left(n\right)},Kb^{\left(n\right)}-Ku^{\left(n\right)}\rangle \\ \geq \alpha {\left\|b^{\left(n\right)}-u^{\left(n\right)}\right\|}_{2}^{2}+\beta {\left\|Kb^{\left(n\right)}-v^{\left(n\right)}\right\|}_{2}^{2}-t\beta {\left\|K\right\|}_{2}{\left\|Kb^{\left(n\right)}-v^{\left(n\right)}\right\|}_{2}{\left\|b^{\left(n\right)}-u^{\left(n\right)}\right\|}_{2}\\ \geq (1-\frac{t\sqrt{\beta }\left\|K\right\|}{2\sqrt{\alpha }})(\alpha {\left\|b^{\left(n\right)}-u^{\left(n\right)}\right\|}_{2}^{2}+\beta {\left\|Kb^{\left(n\right)}-v^{\left(n\right)}\right\|}_{2}^{2}). \end{array}$$

The last inequality in (A6) holds by using $2ab\leq \sigma a^{2}+b^{2}/\sigma$ for any a,b and any σ>0. We choose $\sigma =\sqrt{\alpha /\beta }$ here, and the proof is completed. □

## Appendix C. Proof of Theorem 1

**Proof.** According to the updated Formulae (12) and (13) in Algorithm 1, we obtain

(A7) $$\begin{array}{l} {\left\|b^{\left(n+1\right)}-b^{*}\right\|}_{2}^{2}+{\left\|y^{\left(n+1\right)}-y^{*}\right\|}_{2}^{2}\\ ={\left\|b^{\left(n\right)}-b^{*}\right\|}_{2}^{2}+{\left\|y^{\left(n\right)}-y^{*}\right\|}_{2}^{2}-2\gamma_{\left(n\right)}(\langle b^{\left(n\right)}-b^{*},d_{1}{}^{\left(n\right)}\rangle \\ +\langle y^{\left(n\right)}-y^{*},d_{2}{}^{\left(n\right)}\rangle )+\gamma_{\left(n\right)}{}^{2}({\left\|d_{1}{}^{\left(n\right)}\right\|}_{2}^{2}+{\left\|d_{2}{}^{\left(n\right)}\right\|}_{2}^{2})\\ \leq {\left\|b^{\left(n\right)}-b^{*}\right\|}_{2}^{2}+{\left\|y^{\left(n\right)}-y^{*}\right\|}_{2}^{2}-2\gamma_{\left(n\right)}(\alpha {\left\|b^{\left(n\right)}-u^{\left(n\right)}\right\|}_{2}^{2}\\ +\langle Kb^{\left(n\right)}-v^{\left(n\right)},\beta K{\hat{y}}^{\left(n\right)}-\beta v^{\left(n\right)}\rangle )+\gamma_{\left(n\right)}{}^{2}({\left\|d_{1}{}^{\left(n\right)}\right\|}_{2}^{2}+{\left\|d_{2}{}^{\left(n\right)}\right\|}_{2}^{2}). \end{array}$$

Combining the definition of $\gamma_{\left(n\right)}$ in Equation (14), we have

(A8) $$\begin{array}{l} -2\gamma_{\left(n\right)}(\alpha {\left\|b^{\left(n\right)}-u^{\left(n\right)}\right\|}_{2}^{2}+\langle Kb^{\left(n\right)}-v^{\left(n\right)},\beta K{\hat{y}}^{\left(n\right)}-\beta v^{\left(n\right)}\rangle )+\gamma_{\left(n\right)}{}^{2}({\left\|d_{1}{}^{\left(n\right)}\right\|}_{2}^{2}+{\left\|d_{2}{}^{\left(n\right)}\right\|}_{2}^{2})\\ =-(2-\theta )\gamma_{\left(n\right)}(\alpha {\left\|b^{\left(n\right)}-u^{\left(n\right)}\right\|}_{2}^{2}+\langle Kb^{\left(n\right)}-v^{\left(n\right)},\beta K{\hat{y}}^{\left(n\right)}-\beta v^{\left(n\right)}\rangle ). \end{array}$$

By (A7) and (A8), together with Lemma 1, we further obtain

(A9) $$\begin{array}{l} {\left\|b^{\left(n+1\right)}-b^{*}\right\|}_{2}^{2}+{\left\|y^{\left(n+1\right)}-y^{*}\right\|}_{2}^{2}={\left\|b^{\left(n\right)}-b^{*}\right\|}_{2}^{2}+{\left\|y^{\left(n\right)}-y^{*}\right\|}_{2}^{2}\\ -(2-\theta )\gamma_{\left(n\right)}(\alpha {\left\|b^{\left(n\right)}-u^{\left(n\right)}\right\|}_{2}^{2}+\langle Kb^{\left(n\right)}-v^{\left(n\right)},\beta K{\hat{y}}^{\left(n\right)}-\beta v^{\left(n\right)}\rangle )\\ \leq {\left\|b^{\left(n\right)}-b^{*}\right\|}_{2}^{2}+{\left\|y^{\left(n\right)}-y^{*}\right\|}_{2}^{2}\\ -(2-\theta )\gamma_{\left(n\right)}(1-\frac{t\sqrt{\beta }\left\|K\right\|}{2\sqrt{\alpha }})(\alpha {\left\|b^{\left(n\right)}-u^{\left(n\right)}\right\|}_{2}^{2}+\beta {\left\|Kb^{\left(n\right)}-v^{\left(n\right)}\right\|}_{2}^{2}). \end{array}$$

Therefore, if $1-\frac{t\sqrt{\beta }\left\|K\right\|}{2\sqrt{\alpha }}\geq 0$ and 0<θ<2, it is not difficult to confirm that $\gamma_{\left(n\right)}$ is bounded below by some positive number. Consequently, by the above derivation in (A9), we obtain that the limits of monotonically decreasing nonnegative sequence $\left\{(b^{\left(n\right)},y^{\left(n\right)})-(b^{*},y^{*})\right\}$ exists, thus the sequence $\left\{(b^{\left(n\right)},y^{\left(n\right)})\right\}$ is bounded and

(A10) $${\left\|b^{\left(n\right)}-u^{\left(n\right)}\right\|}_{2}\to 0,{\left\|Kb^{\left(n\right)}-v^{\left(n\right)}\right\|}_{2}\to 0.$$

According to the boundedness of sequence $\left\{(b^{\left(n\right)},y^{\left(n\right)})\right\}$, we know that some subsequence $\left\{(b^{\left(n_{l}\right)},y^{\left(n_{l}\right)})\right\}$ converges to some limit $\left(b^{\infty },y^{\infty }\right)$ (strong convergence due to the dimension of the space being finite). Combing (A10) with the boundedness of the operator K implies that $\lim_{n}K{\hat{y}}^{\left(n\right)}-v^{\left(n\right)}=0$, $\lim_{n_{l}}u^{\left(n_{l}\right)}=b^{\infty }$, $\lim_{n_{l}}\beta (K{\hat{y}}^{\left(n_{l}\right)}-v^{\left(n_{l}\right)})+y^{\left(n_{l}\right)}=y^{\infty }$. From (15), we further obtain

(A11) $$\begin{array}{l} \left(\begin{array}{c} \alpha b^{\left(n\right)}-\alpha u^{\left(n\right)}-K^{T}y^{\left(n\right)}+\beta K^{T}(y^{\left(n\right)}+\beta K{\hat{y}}^{\left(n\right)}-\beta v^{\left(n\right)})\\ v^{\left(n\right)}-Ku^{\left(n\right)} \end{array}\right)\in \\ \left(\begin{array}{c} \partial G(u^{\left(n\right)})+\beta K^{T}(y^{\left(n\right)}+\beta K{\hat{y}}^{\left(n\right)}-\beta v^{\left(n\right)})\\ \partial b(Ku^{\left(n\right)})-(y^{\left(n\right)}+\beta K{\hat{y}}^{\left(n\right)}-\beta v^{\left(n\right)}) \end{array}\right). \end{array}$$

When taking the limit along $n_{l}$, (A11) means

$$\left(\begin{array}{c} 0\\ 0 \end{array}\right)\in \left(\begin{array}{c} \partial G(b^{\infty })+\beta K^{T}(y^{\infty })\\ \partial b(Kb^{\infty })-y^{\infty } \end{array}\right).$$

This means that $\left(b^{\infty },y^{\infty }\right)$ is a critical point of the objective function. Together with the fact that the limit of $\left\{(b^{\left(n\right)},y^{\left(n\right)})-(b^{\infty },y^{\infty })\right\}$ exists, so its limit is also equal to $\left(b^{\infty },y^{\infty }\right)$, i.e., $\lim_{n}b^{\left(n\right)}=b^{\infty },\lim_{n}y^{\left(n\right)}=y^{\infty }(n\to \infty ).$ □
